# From Mesh to Modern Therapies: An Updated Narrative Review on Urogenital Prolapse

**DOI:** 10.3390/jcm14228254

**Published:** 2025-11-20

**Authors:** Diana Pop-Lodromanean, Radu Chicea, Dan-Georgian Bratu, Livia-Mirela Popa, Paula Anderco, Nicolae Grigore, Adrian Hașegan

**Affiliations:** Faculty of Medicine, Lucian Blaga University of Sibiu, 550169 Sibiu, Romania; didi_lodro@yahoo.com (D.P.-L.); radu.chicea@ulbsibiu.ro (R.C.); dan.bratu@ulbsibiu.ro (D.-G.B.); nicolae.grigore@ulbsibiu.ro (N.G.); adrian.hasegan@ulbsibiu.ro (A.H.)

**Keywords:** urogenital prolapse, pelvic organ prolapse, sacrocolpopexy, mesh complications

## Abstract

Urogenital prolapse (UP), a manifestation of pelvic organ prolapse (POP), is prevalent and burdensome, impairing urinary, bowel, sexual and psychosocial health. This review synthesizes evidence on epidemiology, mechanisms, clinical evaluation and treatment, with an emphasis on mesh use. POP results from failure of muscular and fascial support, most consistently associated with childbirth and aging; imaging links levator ani avulsion and hiatal overstretching to onset and recurrence. Diagnosis is chiefly clinical, using standardized pelvic examination, with selective adjuncts such as urodynamics, cystoscopy, pelvic floor ultrasound and defecography. Conservative care includes education, lifestyle measures, pelvic floor muscle training and pessaries. Surgery is considered for bothersome prolapse and individualized by compartment, symptoms, sexual goals, comorbidities and preference. Options span native-tissue vaginal repairs with apical suspension, obliterative procedures for non-sexually active patients and sacrocolpopexy. Sacrocolpopexy remains the durability benchmark for apical support but carries mesh-related risks that accumulate over time. Regulatory scrutiny followed rising complications, culminating in withdrawal of transvaginal mesh kits for anterior prolapse, while mesh for sacrocolpopexy persists. Quality-of-life outcomes are central to assessment. Pain after mesh may reflect placement or evolution (erosion, proximity) or persist despite normal findings, implicating neuroplastic mechanisms. Individualized, shared decision-making is essential to balance durability, safety and function.

## 1. Introduction

Pelvic organ prolapse (POP) is a frequent gynecological disorder, estimated to affect nearly half of women beyond the fourth decade of life [1]. It occurs when the weakening of the pelvic floor muscles, most commonly due to childbirth and aging, allows adjacent organs to descend into the vaginal canal, resulting in prolapse [2]. Given that nearly one-third of prolapse surgeries address recurrent cases, a deeper understanding of the pathophysiology of POP is essential [3,4]. Although its etiology is multifactorial, childbirth remains the most consistent risk factor identified in epidemiological research [5,6]. Emerging imaging evidence implicates levator ani trauma as the crucial link between vaginal delivery and POP, with both levator avulsion (“macrotrauma”) and irreversible hiatus overstretching (“microtrauma”) serving as strong predictors of prolapse onset and postoperative recurrence [7,8,9].

Urogenital prolapse (UP), a prevalent and often distressing component of POP, has a well-documented negative impact on health-related quality of life [10]. It represents the subset of POP that involves the urinary and genital organs, such as the uterus, vaginal vault, urethra and bladder. The condition may involve one or multiple anatomical compartments, including the following:

- *Anterior compartment prolapse*—descent of the bladder, referred to as cystocele;
- *Posterior compartment prolapse*—protrusion of the rectum into the vaginal wall, known as rectocele;
- *Apical compartment prolapse*—descent of the vaginal apex, frequently associated with enterocele;
- *Enterocele*—herniation of small bowel loops into the upper vagina;
- *Uterine procidentia*—complete uterine descent involving all three compartments [11,12].

Modern terminology favors “anterior” and “posterior vaginal wall prolapse” over “cystocele” and “rectocele,” as vaginal wall anatomy does not always mirror the displaced organ. Moreover, because the vagina functions as a continuous structure, compartmental distinctions are somewhat arbitrary; prolapse in one area commonly coexists with descent in another. Notably, nearly half of anterior prolapse cases occur alongside apical descent [13].

Despite this, clinical examinations reveal some degree of prolapse in nearly 40–60% of parous women, many of whom remain asymptomatic. Surgical intervention rates range from 10 to 30 per 10,000 women, a figure expected to rise as the population ages [10,14]. Despite anterior compartment defects being the most commonly documented, accumulating evidence identifies deficient apical vaginal support as a principal contributor to both anterior and posterior compartment descent and as a driver of postoperative recurrence when vault stability is lacking.

From a biomechanical perspective, POP can be considered a form of herniation of pelvic viscera, such as the uterus, bowel or bladder through the levator hiatus, bordered anteriorly by the pubic bone and laterally and posteriorly by the levator ani complex [4]. Disruption of this muscular structure leads to hiatus enlargement and loss of organ support, while fascial injury, particularly that accompanying levator avulsion, further contributes to the pathophysiology, a mechanism especially evident in posterior compartment prolapse [15].

Clinical manifestations include pelvic pain, sensations of pressure or heaviness and the perception or visualization of a bulge protruding through the vaginal canal [16]. Beyond these physical symptoms, it can profoundly affect mental health, social functioning, sexual health and overall quality of life [17]. Given its high prevalence, POP and its management exert a considerable burden on both society and healthcare systems [18]. The most significant risk factors include vaginal delivery and enlargement of the levator hiatus, with additional associations reported for age, birthweight, and body mass index [19].

A strong bidirectional association exists between POP and stress urinary incontinence (SUI), with more than 60% of affected women presenting both conditions [20]. This overlap is attributed to shared etiological factors that contribute to generalized pelvic floor weakness [21]. Women with POP or urinary incontinence consistently report poorer sexual function, as reflected by significantly lower PISQ scores and reduced sexual frequency and satisfaction compared with unaffected individuals [22]. Fear of urine leakage often leads to sexual avoidance and those experiencing both POP and SUI demonstrate a greater decline in libido, arousal and orgasmic satisfaction than women with incontinence alone [23]. The coexistence of SUI is observed in approximately 55% of women with stage II prolapse but decreases to about 33% in stage IV, likely due to urethral kinking or obstruction [24]. In some cases, SUI becomes evident only when the prolapse is reduced, so-called occult SUI, which may be identified in up to 80% of otherwise continent women [12,25,26]. Furthermore, de novo SUI can develop following surgical repair of prolapse in previously continent patients.

This updated narrative review emphasizes developments until 2025, streamlining conventional complications to foreground innovation and clinical relevance.

## 2. Search Strategy and Selection Criteria

This updated narrative review integrates recent evidence identified through structured searches of PubMed, Scopus and Web of Science, with reporting aligned to SANRA guidance for narrative reviews. The primary time window was 1 January 2022 through 30 September 2025, chosen to capture post-2019 regulatory shifts, the introduction of lightweight/macroporous mesh generations and recent trials on regenerative and energy-based therapies. Selective backward searching of pre-2022 seminal studies was performed solely to contextualize long-term outcomes and key regulatory inflection points.

Eligibility criteria included peer-reviewed original research, systematic reviews/meta-analyses and clinically relevant overviews reporting explicit diagnostic criteria, surgical outcomes (including patient-reported measures) or innovation in biomaterials/imaging pertinent to POP/UP. Case reports, letters, non-human studies without translational relevance and papers lacking primary data or explicit methods were excluded.

Two reviewers independently screened titles/abstracts and assessed full texts for eligibility, discrepancies being resolved by discussion. Data extraction captured population, intervention/technology, comparator (when applicable), outcomes, follow-up, and study design, and the evidence was synthesized narratively by domain (epidemiology, clinical evaluation, surgery, mesh/biomaterials, emerging therapies).

We explicitly distinguish technologies in current clinical use (e.g., polypropylene mesh for sacrocolpopexy) from emerging or experimental approaches (e.g., biodegradable scaffolds, regenerative modalities, energy-based devices), highlighted separately to foreground innovation while preserving clinical relevance.

The search used combinations of predefined keywords (“pelvic organ prolapse”, “urogenital prolapse”, “pelvic floor dysfunction”, “mesh complications”, “native tissue repair”, “sacrocolpopexy”), limited to the English-language literature. Reference lists of included articles and major regulatory documents (e.g., FDA, NICE) were also scanned to capture additional eligible sources.

## 3. Epidemiology and General Concepts

Extensive descriptions of the female pelvic floor are available in the existing literature [27,28,29]. In brief, pelvic support is organized into three tiers composed of interlacing musculature and fascial elements:

- *Level I* (cardinal and uterosacral ligaments) suspends the upper vagina and cervix;
- *Level II* (arcus tendineus fascia and pubococcygeus/iliococcygeus fascia) buttresses the mid-vagina against the pelvic wall;
- *Level III* converges at the urogenital diaphragm and perineal body, while the endopelvic fascia provides a variable connective scaffold [27,28,29,30,31,32].

POP arises when this fascial–muscular network loses integrity, permitting descent of pelvic viscera into the vaginal canal [33]. Prevalence estimates for genital prolapse vary widely depending on case definition and ascertainment method. Symptom-based surveys typically report ~2.9–11.4% prevalence, whereas studies using clinical examination with the Pelvic Organ Prolapse Quantification (POP-Q) system have reported ~31.8–97.7% [34,35,36,37,38,39]. Robust population data from many regions in Asia and Africa are lacking because community-based surveys are scarce [40]. Reported U.S. prevalence estimates indicate ~21.7% of women aged 18–83 have prolapse, with higher rates of ~27% and ~30% observed in women aged 30–49 and 50–89 years, respectively; in low-income countries, prevalence is estimated at ~20% [41].

Women may present with prolapse at a single or multiple sites. The etiology is multifactorial, with risk factors such as pregnancy, childbirth, pelvic floor denervation or weakness, congenital or acquired connective tissue defects, aging, menopause, hysterectomy and conditions associated with chronically elevated intra-abdominal pressure [42,43,44]. Although the pathology is multifactorial in origin, multiparity remains one of the strongest risk factors. Pregnancy and vaginal birth stretch and weaken pelvic support structures, while factors such as macrosomia, prolonged labor or instrumental delivery can cause direct muscle or nerve injury, further compromising pelvic floor integrity [45].

Symptoms vary and may include sensations of pelvic heaviness, bulging, dragging or backache, often accompanied by bladder, bowel or sexual dysfunction [46]. Some symptoms are directly linked to the prolapsed organ, such as poor urinary stream with cystocele or obstructed defecation with rectocele, while others, like overactive bladder, may occur independently [47].

Risk factors for symptomatic prolapse include parity and vaginal childbirth, increasing connective tissue disorders, age, elevated body mass index, chronic constipation and menopause, with obesity and constipation representing modifiable contributors that should be addressed in preventive care [6,48,49]. The role of hysterectomy remains uncertain: while one UK cohort subanalysis reported a 5% cumulative incidence of subsequent prolapse surgery over a 15-year period, more recent studies found no increased risk when hysterectomy was performed for non-POP indications [6,50]. Historically, reoperation rates following primary POP surgery were estimated at 30–50%, though contemporary data suggest lower rates of 6–30%, likely reflecting improved surgical techniques and the separate classification of urinary incontinence in outcome reporting [51,52,53,54].

Incorporating apical suspension reduces the likelihood of reoperation [55]. Risk factors for recurrence include age below 60 years at the time of vaginal POP surgery, obesity and advanced preoperative prolapse (stage III–IV) [56,57,58]. The severity is commonly classified according to the maximal extent of organ descent in relation to the hymen. This standardized system, originally proposed by Bump et al. in 1996, defines five stages ranging from no prolapse to complete vaginal vault eversion (Table 1) [59].

## 4. Clinical Evaluation and Therapeutic Approaches

UP is primarily a clinical diagnosis, established through correlation of patient-reported symptoms with physical findings. A comprehensive history should document the duration and severity of symptoms, presence of a vaginal bulge, associated urinary or sexual dysfunction and the degree of impact on quality of life. It often remains asymptomatic until the leading edge descends to the level of the hymen [60,61].

Typical complaints include pelvic heaviness, a sensation of protrusion or visible tissue at the introitus, with symptoms worsening by the end of the day or during physical activity and improving with rest [62]. Compartment-specific manifestations include voiding difficulties or splinting with anterior wall prolapse and incomplete evacuation with posterior defects [60,63].

Sexual dysfunction, most commonly dyspareunia or obstructed intercourse, is also frequent and risk factors should be assessed through a full medical, obstetric and surgical history [62]. Misconceptions that prolapse represents a normal aspect of aging may delay presentation, underscoring the importance of patient education [64].

Physical examination encompasses abdominal and pelvic assessment, beginning with inspection of the vulva and vaginal epithelium for atrophy, ulceration or chronic irritation. Valsalva maneuver or coughing can reveal both prolapse and occult stress incontinence [63]. Palpation of the pelvic floor muscles is essential to evaluate strength and exclude tension myalgia [65].

Speculum examination should systematically assess anterior, apical and posterior compartments, as defects frequently coexist [66]. For documentation, the POP-Q system, an objective, site-specific method that measures prolapse relative to fixed vaginal landmarks, is recommended as the standard, with the Baden–Walker grading system as an alternative [59,67]. POP-Q improves consistency of staging, clinical documentation and comparability of outcomes across studies [60,68].

Additional tests are reserved for specific indications. Postvoid residual measurement and urodynamic studies are useful when voiding dysfunction is present or when occult stress incontinence is suspected in surgical candidates [69]. Cystoscopy is indicated in cases of hematuria or suspected mesh complications, while dynamic imaging (e.g., defecography, anal manometry) may help assess concomitant bowel disorders. Pelvic floor ultrasound is increasingly applied in recurrent cases to detect levator ani avulsion and hiatus ballooning [70,71].

For women without symptoms, education and reassurance typically suffice, since some may not realize that urinary or bowel dysfunction can be attributable to POP. Lifestyle modifications can alleviate symptoms, such as fiber supplementation and osmotic laxatives for defecatory dysfunction or adopting positions that reduce bulge symptoms. Pelvic floor muscle training, performed independently or with supervised guidance, can alleviate symptoms and may help slow further progression [72,73,74]. Evidence for local or systemic estrogen in preventing or treating POP is limited, though topical estrogen may help relieve vaginal irritation [75].

For symptomatic cases, vaginal pessaries represent an effective nonsurgical option, with up to 92% of women successfully fitted [76]. Ring pessaries are most effective for stage II and III prolapse, while advanced cases often require Gellhorn pessaries [77]. Patients should be encouraged to manage pessary care independently when possible; otherwise, regular follow-up is required. Although complications such as erosion (2–9%), ulceration or, rarely, fistula may occur, these are typically manageable with temporary removal, topical estrogen or device adjustment [78,79].

**Figure 1:**
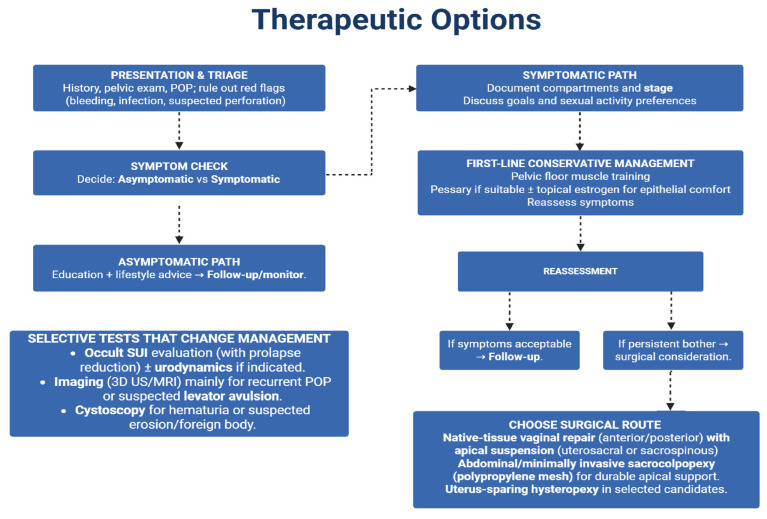
Therapeutic options in pelvic organ prolapse.

Surgery is indicated when prolapse causes significant bother and nonsurgical measures fail or are declined. Both vaginal and abdominal routes are options, with selection guided by the prolapse’s site and severity and the pattern of accompanying symptoms (urinary, bowel or sexual dysfunction), patient comorbidities and preferences and the surgeon’s expertise [80]. Figure 1 shows a chronological therapeutic pathway for pelvic organ prolapse.

## 5. Surgical Management

Surgical management is indicated in women with symptomatic disease who have not achieved adequate relief from or decline, conservative measures. Surgical options are broadly divided into reconstructive procedures, which restore support while preserving the vaginal canal and obliterative procedures, which close the vaginal canal [81]. The choice between these approaches depends on the compartments involved, the severity of prolapse, patient comorbidities, durability of repair and patient preference, with shared decision-making being essential.

Vaginal reconstructive surgery (native-tissue repair) can be performed for anterior, apical or posterior prolapse of any stage. Apical suspension, either to the uterosacral or sacrospinous ligaments, is central when apical defects are present, with randomized trials showing comparable outcomes between these techniques over five years of follow-up [53]. Anterior colporrhaphy is performed for cystocele, while posterior colpoperineorrhaphy addresses rectocele and often includes perineal body reconstruction [82]. Long-term data support good durability, with reoperation-free survival rates of 94% at 5 years and 81% at 10 years [83,84].

Obliterative surgery (colpocleisis) is appropriate for women with advanced prolapse who no longer desire vaginal intercourse [85]. This procedure involves reapproximating the anterior and posterior vaginal walls after epithelial excision [86]. It is highly effective, with success rates between 91 and 100%, fewer complications and lower surgical risk, making it particularly suitable for medically complex patients [63]. Variants include LeFort colpocleisis for women with an intact uterus and total colpocleisis for those post-hysterectomy [87,88,89].

Abdominal or minimally invasive reconstructive surgery (sacrocolpopexy) remains the gold standard for apical prolapse repair, as numerous studies have shown that the use of polypropylene mesh in this procedure yields significantly lower recurrence rates compared with other mesh types or transvaginal repair techniques [14,90,91]. This technique uses a Y-shaped polypropylene mesh to suspend the vagina to the sacrum, most often performed laparoscopically or robotically. Sacrocolpopexy offers superior durability compared with native-tissue vaginal repairs but carries mesh-related risks, including vaginal mesh exposure leading to spotting, pain or dyspareunia [51,92].

Complications following sacrocolpopexy tend to accumulate over time, with mesh exposure reported in 10.5% of cases at seven years, while rates after transvaginal placement are approximately twice as high [12,51]. Documented complications include vaginal wall exposure, erosion into adjacent organs, infection and pain, prompting the U.S. Food and Drug Administration (FDA) to issue two public safety statements: the first in 2008 highlighting the severity of adverse events linked to transvaginal mesh and the second in 2011 emphasizing that such complications are not rare, citing 2874 reports recorded in the MAUDE database over a three-year period [93,94].

Transvaginal polypropylene mesh moved from early enthusiasm to major controversy. After FDA clearance in 1996 it saw rapid adoption for POP but rising complications: Protogen’s 1999 recall, early-2000s litigation and thousands of adverse-event reports by 2010 led to high-risk reclassification in 2011 and requirements for long-term safety/efficacy data in 2012 [17,95]. Since then, regulators including NICE have imposed restrictions or bans, and only a limited number of products remain on the market under strict oversight [17].

Uterus-sparing approaches (hysteropexy) are increasingly studied as alternatives to hysterectomy in well-selected women [96]. These can be performed transvaginally or via sacrocolpopexy, with the choice guided by patient preference, comorbidities and reproductive or personal considerations. Long-term data remain limited [62,97].

Mesh considerations have been subject to significant regulatory scrutiny. FDA mandated the market withdrawal of transvaginal mesh kits used for anterior compartment prolapse because of high complication rates in April 2019 [11]. This ban does not apply to mesh used in sacrocolpopexy or midurethral slings, which remain available but still carry risks of mesh-related complications. For women already implanted with transvaginal mesh who are asymptomatic, no intervention is recommended; however, persistent bleeding, discharge, pelvic pain or dyspareunia should prompt clinical evaluation [11,62].

Recent research has provided valuable insights into the multifactorial nature, diagnostic assessment and management of urogenital prolapse. A growing body of evidence highlights the interplay between obstetric history, connective tissue integrity and surgical technique in determining both the onset and recurrence of prolapse (Table 2).

## 6. Emerging and Next-Generation Therapies

In current practice, polypropylene mesh for sacrocolpopexy remains the durability benchmark [104]. Technologies are categorized into current clinical use: for example, abdominal polypropylene mesh for sacrocolpopexyand investigational approaches, including biodegradable scaffolds, regenerative modalities and energy-based devices [105,106]. Evidence for the latter is summarized to contextualize innovation and should not be interpreted as clinical equivalence to established surgical standards. Biodegradable scaffolds, regenerative therapies and energy-based devices are investigational and still lack robust anatomic outcome data, and no pharmacologic therapy reverses POP [107,108].

In parallel with advances in mesh design and surgical technique, several next-generation strategies are under investigation to improve durability and biocompatibility in POP repair [109]. Biodegradable meshes based on poly (ε-caprolactone) (PCL), including melt-electrowritten (MEW) designs that mimic vaginal tissue mechanics, have demonstrated favorable in vitro/in silico performance and manufacturability, aiming to reduce chronic inflammation and late complications seen with permanent polypropylene [110,111,112].

Recent MDPI studies report auxetic or antistatic MEW-PCL architectures that maintain porosity and strength while supporting tissue integration, highlighting a translational path toward resorbable POP implants; however, clinical data remain absent [110,111]. Simulation-validated PCL “cog threads” have been proposed as a minimally invasive reinforcement concept for the vaginal wall, illustrating the breadth of biodegradable approaches under evaluation [113].

Preclinical and early translational research suggests mesenchymal stromal cell-based therapies and mesenchymal stromal cell derived exosomes may modulate extracellular-matrix remodeling and pelvic connective-tissue repair in pelvic floor dysfunction, including POP, but human efficacy data specific to prolapse correction are not yet established [114,115]. No pharmacologic agent has shown an ability to reverse anatomical prolapse; current medical therapy is primarily symptomatic (e.g., genitourinary syndrome of menopause) or supportive for pessary care [109].

In a multicenter randomized, double-blind, placebo-controlled trial, intravaginal estrogen did not improve 12-month pessary continuation with satisfaction versus placebo, although adverse events were reduced [116]. This informs counseling on symptom relief but does not constitute an anatomic treatment for prolapse. Perioperative topical estrogen around native-tissue prolapse surgery did not improve long-term surgical outcomes versus placebo in a randomized superiority trial, underscoring the limited role of pharmacologic therapy for anatomic success [117].

Two randomized studies in 2024–2025 evaluated transvaginal temperature-controlled radiofrequency for SUI, showing symptomatic improvements versus comparators; these results pertain to continence endpoints rather than anatomical POP correction. Given the heterogeneity of devices and protocols and the focus on SUI or vaginal laxity, EBDs should be considered investigational for POP until trials with anatomical outcomes and long-term safety are available [118,119].

## 7. Mesh Implants

Since the introduction of the tension-free vaginal tape in 1998, synthetic and biological materials have become established in urogynecological surgery [120]. While nearly 11% of women undergo prolapse surgery and about 30% require reoperation within four years due to failure of native tissue repair, the use of mesh materials was introduced with the aim of improving long-term outcomes [121]. Since 2002, the FDA has cleared approximately 60 mesh procedures for prolapse repair through the 510 (k) exemption pathway, a process that allows market entry without comprehensive clinical evidence [11,122]. The reference device for these applications was a hernia mesh.

In current practice, polypropylene mesh for sacrocolpopexy remains the durability benchmark, whereas transvaginal mesh kits for anterior repair are no longer marketed following regulatory actions [123,124]. By contrast, biological/composite grafts have not shown consistent superiority in durability or cost-effectiveness and biodegradable scaffolds remain investigational [125,126].

Implantation of mesh invariably triggers a foreign body reaction characterized by macrophage and giant cell infiltration into the mesh pores, generating a chronic inflammatory response and formation of an internal granuloma [127,128]. This process results in encapsulation by an external fibrotic capsule. When pore size is small (<1 mm), scar tissue fills the inter-fiber space and subsequent contraction promotes shrinkage, deformation, and stiffness of the mesh [100,101]. In contrast, larger pores (>1 mm) allow infiltration by local tissue, often fat, thereby preserving flexibility and elasticity. Meshes with very small pores (<1 mm) or multifilament structures hinder macrophage penetration, impairing local defense against infection, an especially relevant concern in the contaminated vaginal environment, thereby increasing the risk of infection and mesh erosion [100,101].

A variety of implantable materials have been explored in the surgical repair of POP and UP, broadly divided into synthetic, biological and composite categories [129,130]. Synthetic meshes, most notably polypropylene, have historically dominated clinical practice due to their affordability, ease of use and mechanical strength [131]. In contrast, biological grafts, whether autologous or heterologous, were developed with the aim of improving biocompatibility and reducing foreign body reactions [120,129,132,133]. However, their clinical performance has been limited by high recurrence rates and elevated costs. Composite meshes combine absorbable and non-absorbable fibers or incorporate coatings (e.g., porcine collagen) in an effort to improve tissue integration and reduce complications, though their clinical performance remains inconsistent [122]. Table 3 provides a comparative overview of the advantages and disadvantages of synthetic versus biological mesh implants.

## 8. Complications of Mesh Use

Over the past 10–15 years, rising mesh-related complication rates have necessitated weighing the higher upfront costs of lighter meshes against their potential to reduce short- and long-term adverse outcomes, a cost-effectiveness challenge that is particularly acute in developing and mid-development settings, including parts of Europe [134].

Quality of life should be tracked longitudinally, as early symptom relief can be offset by late complications or recurrence, using validated instruments such as the Pelvic Floor Impact Questionnaire and Pelvic Floor Distress Inventory [135].

Evidence consistently shows improvements in physical symptoms, mental well-being and sexual function, particularly reduced dyspareunia and greater sexual satisfaction, though results vary by technique and patient characteristics [12,136]. Nonetheless, long-term monitoring remains essential, as recurrence and complications may compromise quality-of-life gains.

Additional complications include chronic pain, infection, dyspareunia, mesh shrinkage, organ perforation and prolapse recurrence, all of which can markedly impair quality of life [131]. Pain after mesh implantation tends to follow two patterns: from abnormal placement/evolution or despite normal placement and evolution [137]. In the first, pain is usually due to organ injury/erosion or nerve entrapment (pudendal, obturator, ilioinguinal) and may also reflect mesh contraction, retraction, thickening or nodularity, often presenting as dyspareunia [137,138]. Evaluation should be symptom-guided and include focused examination, cystoscopy when indicated and pelvic-floor imaging, with 2D/3D ultrasound particularly useful for detecting contraction or nodules and cross-sectional imaging added as needed [139,140].

Limited exposure or localized symptoms may respond to conservative care or partial excision, whereas persistent exposure, organ compromise or refractory pain often warrant removal [141,142]. In the second pattern, investigations are typically normal: mesh removal seldom improves symptoms and can worsen them and up to 40% of patients report pelvic pain predating implantation, suggesting non-implant drivers [138].

Pre-existing pelvic trauma can predispose individuals to chronic pain independent of implant status, aligning with observations that a substantial proportion of patients report pelvic pain before mesh implantation and supporting a multidisciplinary approach [143,144,145,146,147].

## 9. Current Controversies and Research Gaps

Despite major advances in the understanding and management of urogenital prolapse, several key controversies and knowledge gaps persist. The most debated issues concern the long-term safety and durability of synthetic versus biological meshes, the role of uterus-sparing procedures and the lack of standardized outcome reporting across clinical studies [96,134,148].

Reported outcomes vary with study design (randomized vs. observational), follow-up duration, and endpoint definitions (anatomic vs. symptomatic vs. composite), which limits cross-study comparability [149]. Multiple evaluations of prolapse trials highlight inconsistent outcome selection and suboptimal reporting, underscoring the need for harmonization [150]. Standardization should combine POP-Q-based anatomic thresholds with validated patient-reported measures, as recommended by international societies, and be supported by core outcome sets and ≥5–10-year follow-up to capture durability and late adverse events [59,151].

Long-term follow-up data for newer lightweight and macroporous mesh systems remain limited, with most studies reporting outcomes within 3–5 years post-surgery [100,152]. While some trials demonstrate lower recurrence and exposure rates compared with earlier mesh generations, heterogeneity in study design, patient selection and reporting metrics precludes definitive conclusions [148,153,154,155]. Furthermore, mesh-related complications such as chronic pain or dyspareunia continue to generate medico-legal and ethical debate, particularly regarding informed consent and device surveillance [134].

Another unresolved question involves the optimal management strategy for younger and sexually active women. Balancing anatomical durability with functional and sexual outcomes remains challenging, as reconstructive procedures may preserve vaginal function at the expense of higher recurrence rates, whereas obliterative approaches, though safer, eliminate coital function entirely [156,157,158,159].

Significant knowledge gaps also exist in understanding the biological mechanisms underlying recurrence. The interplay between levator ani avulsion, connective tissue remodeling, hormonal status, and genetic predisposition is increasingly recognized but insufficiently characterized [160,161,162]. Multi-omic approaches integrating genomics, proteomics, and imaging biomarkers could elucidate individualized risk profiles and guide tailored preventive strategies.

Finally, inconsistencies in defining surgical success, whether anatomical correction, symptom relief or quality-of-life improvement, limit cross-study comparability [150,163,164]. The absence of harmonized reporting frameworks underscores the urgent need for standardized core outcome sets in prolapse research. Collaborative, multicenter registries with long-term follow-up would provide higher-quality data to inform evidence-based guidelines. A comparative summary of procedures and materials with key outcome signals is provided in Table 4 [12,14,51,63,82,83,84,85,86,87,88,89,90,91,92,122,123].

## 10. Future Perspectives and Conclusions

The management of urogenital prolapse is entering a new era shaped by the convergence of biomaterial innovation, regenerative medicine and precision imaging. Traditional surgical approaches, while effective in restoring anatomy, often fail to address the underlying pathophysiology, particularly the connective tissue degeneration and neuromuscular dysfunction that predispose individuals to recurrence. Future strategies must therefore move beyond mechanical correction toward functional regeneration of pelvic support structures.

One major direction involves personalized surgical planning using dynamic pelvic imaging modalities such as 3D ultrasound and high-resolution MRI, which allow for precise visualization of levator ani avulsion, fascial defects and apical support loss. These technologies can guide individualized surgical selection, whether that is native tissue repair, mesh-augmented reconstruction or regenerative scaffold placement, based on each patient’s unique anatomical and functional profile.

At the biomaterial level, research is rapidly evolving toward biodegradable, immunomodulatory scaffolds that integrate with host tissues, stimulate angiogenesis and degrade synchronously with collagen remodeling. Hybrid scaffolds incorporating extracellular matrix components or growth factors show promise in reducing chronic inflammation and improving long-term durability.

Parallel developments in cell-based regenerative therapy, including mesenchymal stem cells, exosome-enriched hydrogels and gene-modified fibroblasts, offer potential to restore biomechanical function through controlled tissue regeneration rather than passive reinforcement. These biologically active approaches could transform the long-term outcomes of pelvic reconstruction, particularly in younger or high-risk patients where native tissue integrity is critical.

A second key priority for the coming decade is standardization of outcome measurement. Current studies variably report anatomical success, symptom relief or quality-of-life improvement, creating major barriers to meta-analytic synthesis. The adoption of core outcome sets and validated patient-reported measures will be essential for establishing comparability across trials.

Long-term prospective registries and multicenter studies are urgently needed to monitor device safety, validate regenerative therapies, and assess the real-world impact of emerging technologies. Biomaterial innovation should advance within explicit regulatory and ethical guardrails. Following the FDA’s market withdrawal of transvaginal mesh for POP and earlier safety communications, future device development requires stronger pre-market evidence and post-market surveillance.

Informed consent must transparently address uncertainties in long-term durability and potential effects on pain and sexual function, alongside equitable access to revision expertise and robust adverse-event reporting and manufacturer accountability. These data will be pivotal for developing evidence-based international guidelines and ensuring regulatory transparency.

## Figures and Tables

**Table 1:** Staging of POP by the maximal descent in relation to the hymenal plane.

STAGE	DEFINITION	DESCRIPTION
**0**	Without prolapse	All anterior and posterior reference points are at −3 cm, and the cervix or vaginal vault remains well superior to the hymenal plane.
**I**	Mild prolapse	The lowest point of prolapse is more than 1 cm above the hymen.
**II**	Moderate prolapse	The most dependent point lies within the interval from 1 cm above to 1 cm below the hymen.
**III**	Advanced prolapse	The most dependent point descends over 1 cm below the hymen but does not reach the total vaginal length.
**IV**	Complete prolapse (procidentia)	Complete eversion of the vaginal vault is present, with the lowest point protruding to the full vaginal length.

**Table 2:** Recent evidence on urogenital prolapse: epidemiology, risk factors and surgical outcomes.

Author (Year)	Study Type/Population	Focus Area	Main Contributions	Clinical Significance
Schulten et al., 2022 [19]	Systematic review and meta-analysis (*n* = 47,429)	Risk factors for primary and recurrent prolapse	Identified parity, vaginal delivery, BMI, age and levator defects as major determinants of prolapse onset and recurrence	Establishes the multifactorial nature of urogenital prolapse and guides preventive strategies
Vermeulen et al., 2023 [98]	Prospective cohort (16 years post-hysterectomy, *n* = 247)	Pelvic floor symptoms and anatomy after hysterectomy	Found that nearly half of women reported persistent pelvic floor dysfunction regardless of anatomic POP stage	Demonstrates that functional impairment can persist independently of visible prolapse
Loukopoulou et al., 2025 [99]	Cross-sectional (*n* = 134, Greece)	Uterine prolapse prevalence and risk factors	Reported 15.7% prevalence; parity, menopause and age strongly associated with prolapse	Provides region-specific epidemiological data relevant to prevention and early screening
Huang et al., 2025 [100]	Retrospective cohort (*n* = 180; 3-year follow-up)	Calistar mesh repair outcomes	Showed 96% anatomical success with significant improvement in urinary distress and quality of life	Supports the safety and efficacy of new-generation transvaginal mesh systems; non-randomized evidence
Wilczak et al., 2024 [101]	Retrospective observational study	G-Mesh^®^ performance in cystocele/rectocele repair	Demonstrated favorable long-term durability and low complication rates using lightweight macroporous mesh	Confirms progressive design improvements and enhanced patient tolerance of synthetic meshes
Triepels et al., 2024 [102]	Prospective imaging study	MRI predictors of pessary fitting success	Identified total vaginal length and levator orientation as predictors of conservative management success	Strengthens the role of imaging in individualized treatment planning
Edwards et al., 2013 [103]	Review	Mesh-related complications and safety alerts	Summarized early post-market complications including erosion, pain and dyspareunia	Provides historical context for regulatory reforms and mesh technology evolution

**Table 3:** Comparative overview of biological and synthetic mesh implants for surgery.

Category	Materials		Advantages	Disadvantages
**Synthetic**	Absorbable	Polyglactin	- Most widely used material in POP surgery - Readily available and inexpensive compared to biologics - Provides strong and durable mechanical support, ensuring long-term structural reinforcement - Easy handling during surgery due to pre-shaped designs - Can be mass-produced with standardized quality	- Linked to substantial risks of complications, including mesh exposure, erosion, chronic pain and infection. - Once integrated into tissue, removal is technically difficult and may cause further morbidity - Long-term complications (e.g., dyspareunia, nerve entrapment) may persist even after partial excision - Public health controversies and medicolegal implications have reduced acceptance
		Polyglycolic acid		
	Nonabsorbable	Multifilament		
		Monofilament		
	Mixed absorbable/nonabsorbable			
**Biological**	Autologus		- Derived from human or animal tissue, providing better biocompatibility - Potential to reduce long-term foreign body reaction compared with synthetics - Theoretical advantage of improved integration with host tissue - Lower likelihood of immune rejection or severe inflammatory response	- Substantially more expensive than synthetic alternatives - Harvesting autologous grafts requires additional procedures, increasing operative time and donor-site morbidity - Lack of sufficient mechanical durability, with frequent degradation over time - High recurrence rates and treatment failures compared with synthetic meshes - No clear improvement in outcomes compared with native tissue repair, raising questions about cost-effectiveness
	Allograft			
	Xenograft			

**Table 4:** Comparative outcome signals across POP therapeutic procedures and materials.

Procedure/Material	Key Endpoints	Representative Results
Abdominal/minimally invasive sacrocolpopexy (polypropylene mesh)	Anatomic success, recurrence, mesh exposure	Vaginal mesh exposure ~10.5% at 7 years; lower recurrence vs. native repairs and vs. transvaginal mesh techniques
Transvaginal polypropylene mesh kits (anterior repair; withdrawn/restricted)	Exposure/erosion, pain/dyspareunia	Exposure approximately double vs. abdominal placement; subsequent regulatory restrictions/withdrawals
Native-tissue vaginal repairs (with apical suspension when indicated)	Durability (reoperation-free survival), symptoms	Reoperation-free survival ~94% at 5 years; ~81% at 10 years
Colpocleisis (obliterative)	Anatomic success, complications	Success 91–100%, fewer complications and lower surgical risk in medically complex patients
Biological/composite grafts	Recurrence, cost-effectiveness	No consistent superiority to native repair; higher costs
Lightweight/macroporous polypropylene (abdominal)	Exposure, anatomic durability	Trend toward lower exposure vs. dense legacy meshes

## Data Availability

Not applicable.
